# Selective Production of Methanol via Bidentate Coordination of an Intermediate on the Dual‐Atom Electro‐Catalyst

**DOI:** 10.1002/smtd.70874

**Published:** 2026-07-28

**Authors:** Imran Muhammad, Danish Khan, Tanveer Hussain, Khuram Walayat, Awais Ghani, Kemeng Ji, Shehzad Ahmed, Asif Mahmood

**Affiliations:** ^1^ National Industry‐Education Platform of Energy Storage Tianjin University Tianjin People's Republic of China; ^2^ College of New Materials and New Energies Shenzhen Technology University Shenzhen Guangdong China; ^3^ School of Science and Technology University of New England Armidale New South Wales Australia; ^4^ Department of Chemistry University of Warwick Coventry UK; ^5^ Multifunctional Electronic Ceramics Laboratory, College of Engineering Xi'an International University Xi'an People's Republic of China; ^6^ Key Laboratory for Green Chemical Technology of Ministry of Education, Collaborative Innovation Center of Chemical Science and Engineering (Tianjin), School of Chemical Engineering and Technology Tianjin University Tianjin People's Republic of China; ^7^ China‐UK Low Carbon College Shanghai Jiao Tong University Shanghai People's Republic of China; ^8^ Centre for Clean Energy Technology, School of Mathematical and Physical Sciences University of Technology Sydney Sydney New South Wales Australia; ^9^ School of Mathematical and Physical Sciences Faculty of Science, University of Technology Sydney New South Wales Australia; ^10^ Institute of Sustainable Industries and Liveable Cities Victoria University Melbourne Victoria Australia

**Keywords:** *OCHO intermediate, CO_2_RR, constraint molecular dynamics simulations, methanol product, Sn─Cu/C_3_N_4_ catalyst

## Abstract

Electrochemical CO_2_ reduction offers a route to produce liquid fuels such as methanol; however, strong competition from hydrogen evolution and limited control over key reaction intermediates lead to low single‐product selectivity and poor stability under operating conditions. Heteronuclear dual‐atom catalysts (DACs) have shown great promise in this regard because two neighboring catalyst atoms can cooperatively bind and polarize oxygenated intermediates. Despite these attractive features, DACs often struggle to stabilize the right early intermediate for methanol, so CO_2_ protonation defaults back to *COOH (and then *CO), which breaks methanol selectivity. Here, we utilize DAC systems stabilized on a carbon nitride (C_3_N_4_) framework to resolve this mechanistic bottleneck at the molecular level using density functional theory and constrained molecular dynamics simulations. A Sn–N_2_/Cu–N_2_ DAC embedded in a C_3_N_4_ framework, coupled to an explicit aqueous interface and evaluated under an applied potential, is shown to favor methanol formation through a six‐electron *OCHO pathway. The neighboring Sn and Cu sites synergistically stabilize a bidentate *OCHO intermediate through Sn─O p‐orbital interactions, while suppressing formation of *COOH. The results provide a clear design rule for methanol‐selective CO_2_RR: enforce cooperative bidentate binding that locks in *OCHO and redirects the first protonation step away from *COOH and toward methanol.

## Graphical abstract

This study utilizes density functional theory (DFT) and constrained molecular dynamics (cMD) to investigate the free energy landscape of the electrochemical CO_2_ reduction reaction at a liquid–solid interface. The research highlights a dual‐atom catalyst—specifically a Cu─Sn pair anchored in a nitrogen‐doped carbon framework—that facilitates the conversion of CO_2_ into methanol (CH_3_OH) via an OCHO intermediate.

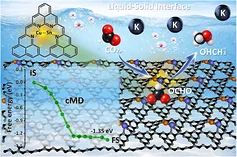

## 1 Introduction

Though electric vehicles generate 17%–30% less carbon dioxide (CO_2_) than gasoline‐ and diesel‐powered engines, carbon emissions from hydrocarbon deposits have risen in 2023 [1]. Nonetheless, the electrocatalytic CO_2_ reduction reaction (CO_2_RR) provides viable pathways for CO_2_ utilization and forges the fastidious process toward valuable carbon‐based chemicals [2, 3]. Considering the C_1_ product of CO_2_ reduction, methanol (CH_3_OH) offers the advantages of facile storage, high energy density, low flammability risk, and transport under atmospheric conditions and is also a valuable feedstock [4, 5]. Methanol is traditionally manufactured through syngas, with a massive amount of CO_2_ (≈1.38 tons of CO_2_ and 0.19 tons of H_2_ are required to produce 1 ton of CH_3_OH); therefore, it is immensely urged that the CH_3_OH production criterion can be diverged by synchronously accomplishing high stability and selectivity [6, 7]. Additionally, the hydrogenation of CO_2_ into CH_3_OH, which involves the transfer of six electrons, typically undergoes high‐energy barriers [8, 9]. There is no doubt that the practical design of an electrocatalyst for CO_2_ reduction to methanol is exceedingly desirable. Owing to the peculiar electronic properties of metal centers and explicit catalysts, single‐atom catalysts (SACs) have attracted significant attention for CO_2_ reduction and conversion into key intermediates (*COOH and *CO) via low‐energy barriers [10, 11, 12, 13]. In addition, SACs, especially Co‐, Ni‐, and Cu‐based systems and MXenes, for efficient CO_2_ reduction to valuable products, emphasizing the roles of support materials [14, 15]. However, SACs with isolated single‐atom sites are inadequate to effectively regulate complicated reaction intermediates, exhibit a lack of selectivity for specific reaction pathways, and are ineffective at preventing undesired side reactions; therefore, certain reactions require adjacent active sites for optimal catalytic performance [16, 17]. Dual‐atom catalysts (DACs) introduce adjacent dual‐atom sites with a degree of freedom for optimizing different catalyzing steps by enabling the microenvironment of active sites compared with SACs, thus elevating the intrinsic properties of catalysts for multifunctional catalytic activities [18, 19]. DACs with analogous atoms, for instance, (Ni, Ni)‐N‐C catalyst form towards the simplistic CO product, and the Cu_2_‐B_x_C_y_ [20] leads to the CO_2_ reduction into *COOH, *CO, and *HCO intermediates [21], while the heteronuclear (Ni, Fe)–N–C dual‐metal single‐atom catalyst, which is based on Ni‐N_4_ and Fe‐N_4_ sites, modulates the atomic environment and tunes the selectivity to CH_3_OH product [22, 23, 24, 25, 26, 27]. Moreover, transition‐metal‐free dual‐atom TCNQ catalysts efficiently activate CO_2_ and selectively reduce it to methanol (CH_3_OH) rather than to other C1 products [28]. CO_2_RR on DACs is expected to yield multi‐carbon products, as the DACs are thought to provide two metal sites for C─C coupling, though C2 products are rarely observed experimentally [29]. However, recent computational screening indicates that DACs are less favorable for C─C coupling and are more energetically active for HCOOH formation [30, 31]. DACs effectively stabilize target intermediates by combining the accuracy of SACs with cooperative effects, thereby providing a product with higher activity, selectivity, and stability.

Here, we report a systematic study of a Sn─Cu dual‐atom C_3_N_4_ catalyst for the selective production of methanol from CO_2_ reduction through a typical bidentate coordinated OCHO* intermediate led by protonation of carbon instead of oxygen, employing the density functional theory (DFT) and constraint molecular dynamics (cMD) in unison, endeavoring the effects of potential and solvation. The free energy for CO_2_ activation follows the correlatively decreasing pattern for accelerating potential, establishing the coordination between the K^+^ ions and the corresponding CO_2_ intermediate. Thereafter, the protonation of CO_2_ favorably forms the *OCHO intermediate while restraining the evolution of *COOH. Furthermore, the necessary role of the Sn atom promotes oxygen coordination and channelizes the OCHOH* intermediate, elucidating the rate‐determining step on the Sn─Cu/C_3_N_4_ catalyst for the production of OHCH_3_. This theoretical exploration unveils new insights into the stabilization of CO_2_ activation under the influence of alkali metal ions, emphasizing the potency of solvation and the potential‐based environment, together with robust electrochemical reaction simulations, which highlight bidentate coordination of primary and secondary transition‐state species and assign the route to methanol production.

## 2 Results

Our core focus is centered on the activation of CO_2_ and the thoroughgoing electrocatalytic reaction for transmutation into methanol (OHCH_3_). The design of the optimized structure of the dual‐atom Sn─Cu/C_3_N_4_ catalyst with dinuclear Sn─Cu active centers is shown in Figure S1. The stability of the Sn─Cu/C_3_N_4_ catalyst is gauged by adsorbing a dual‐atom Sn─Cu in a C_3_N_4_ monolayer, exhibiting a high binding energy of −3.2 eV. Additionally, the previous experimental study on the Sn─Cu/C_3_N_4_ catalyst confirms its stability and applicability to CO_2_RR [31]. The CO_2_ molecule is adsorbed in the gas phase on the Sn─Cu/C_3_N_4_ catalyst to analyze its adsorption and electronic characteristics. Projected density of states (PDOS) results (see Figure S2) reveal significant orbital overlap between CO_2_‐derived C/O *2p* states and Cu *3d*/Sn *5p* states below the Fermi level, indicating electronic coupling and charge redistribution that facilitate CO_2_ activation. Complementary crystal orbital Hamilton population (COHP) analysis confirms bonding interactions between CO_2_ and the Sn─Cu dual‐metal site (see Figure S3), where the C─Cu bond exhibits the strongest contribution (ICOHP = −2.39 eV, bond distance = 2.06 Å); however, the C─Sn, Cu─O, and Sn─O bonds display weaker interactions (ICOHP = −1.33, −1.18, and −1.10 eV, respectively). Additionally, the Cu *d*‐band center shifts from −2.76 to −3.05 eV upon CO_2_ adsorption, as presented in Figure S4, demonstrating a clear modification of the electronic environment. These results confirm that CO_2_ is chemisorbed at the Sn─Cu dual‐metal site through strong electronic interactions rather than weak physisorption.

**FIGURE 1:**
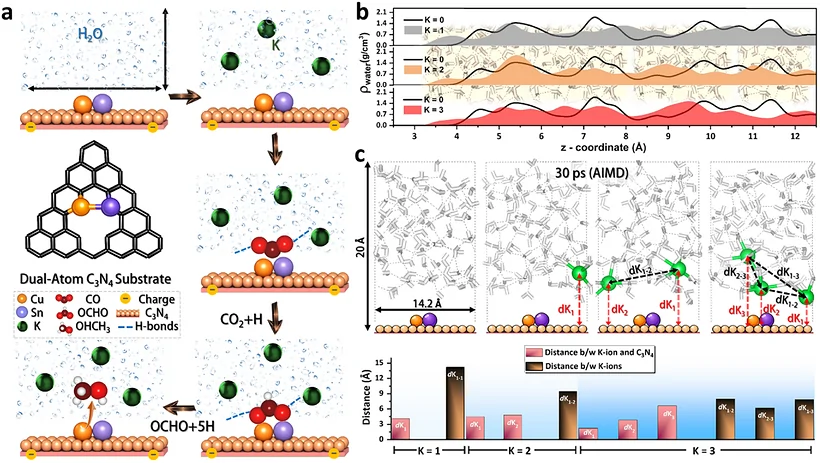
Electrocatalytic mechanism of CO_2_ conversion into methanol and AIMD simulations of Sn─Cu/C_3_N_4_‐K_x_^+^(H_2_O) models: (a)Schematic illustration of the CO_2_RR process on Sn─Cu/C_3_N_4_ catalyst under an aqueous medium and electrode potential by alkali metal ions (AM), where the catalytic reaction path for the hydrogenation of CO_2_ to methanol electroconversion through unconventional *OCHO intermediate. (b) Density distribution of water molecules vertically in the *z*‐direction to the Sn─Cu/C_3_N_4_ models consisting of cation‐free and cation‐containing models, where the Sn─Cu atoms are situated at a distance of 0 Å. (c) Distance between K^+^ cations and Sn─Cu/C_3_N_4_ catalyst in the solvent. Water molecules are shown as white sticks for clarity; K^+^ ions are highlighted as green spheres; Sn is represented as purple, Cu as golden, and the C_3_N_4_ is shown as a reddish‐brown slab. In the corresponding representative plot, the distance between the catalyst and cation is denoted by *dK_x_*, and *dK_x‐y_* denotes the cation–cation (K*_x_*‐K*_y_*) distance. Results are extracted after a 30‐s AIMD simulation at 300 K.

Figure 1a schematically sketches the Sn─Cu dual‐atoms C_3_N_4_ catalyst subjected to solvation and the alkali metal ion's (AM) potential milieu, which conceivably adsorbs the CO_2_ and reduces it into OHCH_3_ with six selective hydrogen exchange reactions through *OCHO intermediates. Previous computational studies of nitrogen reduction have recognized the significance of the electrochemical environment, employing DACs such as Fe_2_, Co_2_, W_2_, and Fe–TM‐based HDAC catalysts to elucidate catalytic energetics and kinetics [32, 33]. In lieu of oft‐repeated *COOH intermediate species from the first hydrogenation reaction, the Sn─Cu dual‐atom C_3_N_4_ catalyst methodically bolsters the OCHO* intermediates. For the simulation scope, we design a model composed of a 2 × 2 × 1 supercell of C_3_N_4_ with a lattice constant of *a* = *b* = 14.20 Å, *c* = 20.0 Å, in the synchronicity of lattice angles: *α* = *β* = 90°, *γ* = 120°. Sn─Cu dual atoms are adsorbed in the pore of C_3_N_4_, and the deuce atoms form two bonds with nitrogen atoms. The void space of 20 Å along the *z*‐direction is brimmed by 88 H_2_O molecules, ostend the 1 atm pressure displayed in Figure S5. In addition, Figure 1b shows the density distribution of water perpendicular to the interface under different potential environment conditions of K^+^‐free and K^+^‐containing aqueous solutions. Different from a pure water system, peaks are observed near the Sn─Cu/C_3_N_4_‐water interface for cation‐containing systems. Evident peaks are noticed away from the Sn─Cu/C_3_N_4_‐water interface, showing a bulk water layer density higher than 1gcm^−3^ for a pure water system. No prominent change in water density is noticed for the K^+^‐containing systems.

On the contrary, we perform simulations to determine the distances between the K^+^‐ions and the Sn─Cu dual‐atom C_3_N_4_ catalyst concealed by a liquid medium indicated by Sn─Cu/C_3_N_4_‐K_x_^+^(H_2_O) systems. Figure 1c illustrates the geometries and corresponding distances of cations in Sn─Cu/C_3_N_4_‐K_x_^+^(H_2_O) systems. In the Sn─Cu/C_3_N_4_‐K_x_^+^(H_2_O) system with a fixed lattice size, the gradual increase of K‐ions involves several electrostatic effects. When the first K‐ion is placed in the lattice, the K‐ion minimizes its repulsive interactions with neighboring K‐ions in a repeated lattice. Considering the K‐ion hydration shell and the electrostatic interaction between ions and the catalyst, it maintains a certain distance from the catalyst. As the number of K‐ions increases, these cations repel each other more effectively, which affects their distribution in the lattice and minimizes the repulsive force. At lower concentrations, water molecules form a stable solvation around K‐ions. However, at high concentrations, water molecules are more structured around the ions. In the Sn─Cu/C_3_N_4_‐K_x_^+^(H_2_O) systems possessing high concentrations of ions, the distance between K‐ions and the catalyst increases because ions tend to minimize the repulsion by spreading out. Overall, a more uniform ionic distribution of K‐ions is caused by the reorganization of ions’ positioning.

### 2.1 Influence of Potassium Ion (K^+^) on CO_2_ Accumulation and Corresponding *COOH and *OCHO Intermediates

**FIGURE 2:**
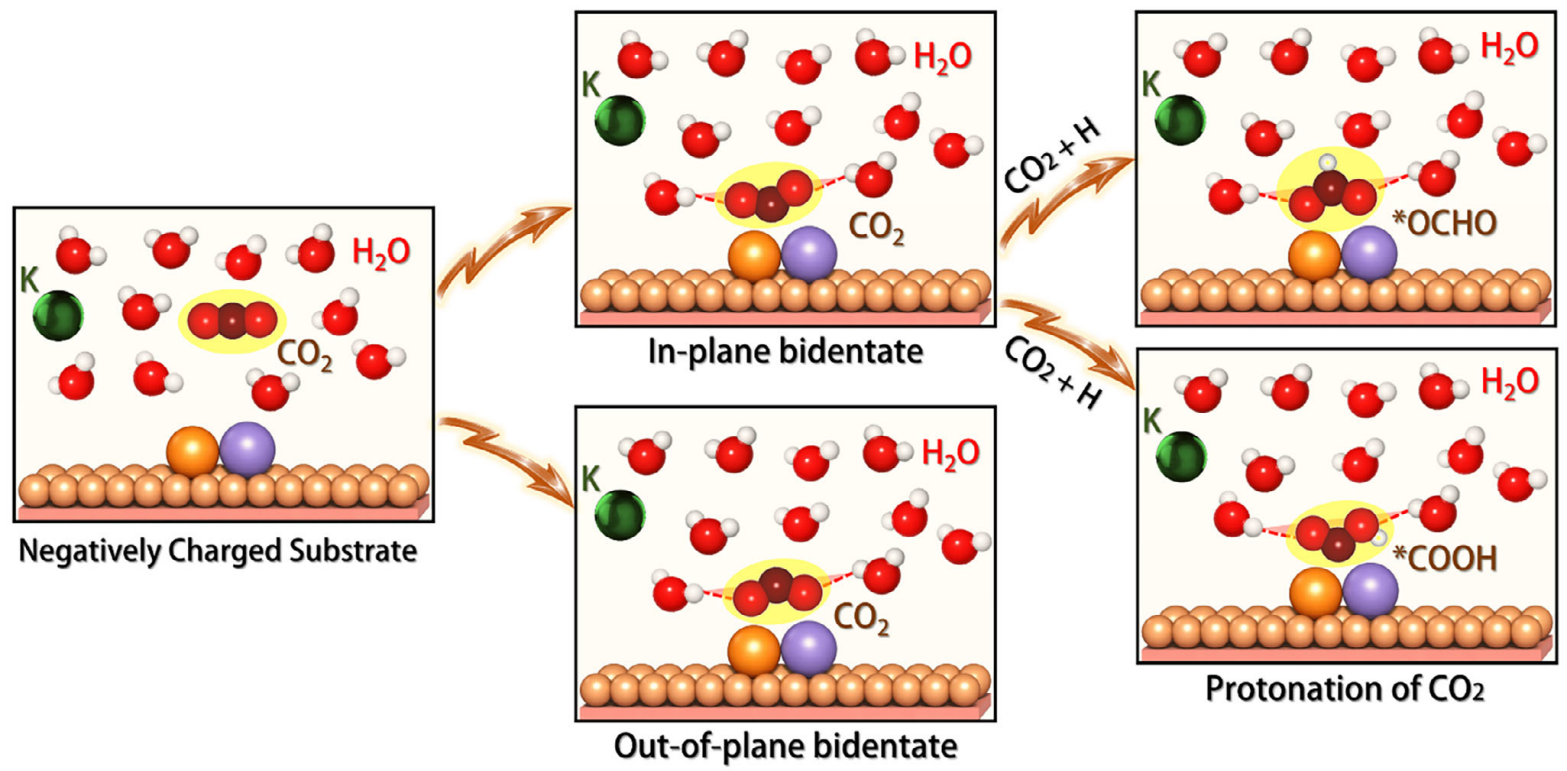
CO_2_ adsorption and response to the first protonation: Schematic illustration of CO_2_RR steps initiating from the stable in‐plane and unstable out‐of‐plane bidentate adsorption of CO_2_ on Sn─Cu/C_3_N_4_ in the presence of cations and liquid medium, the initial hydrogenation of in‐plane bidentate *CO_2_ resulting in monodentate coordinated *COOH and bidentate coordinated *OCHO.

The effect of ions on CO_2_ activation is momentously diversified by ion type and concentration, as well as the involved catalyst. Typically, alkali metal cations stabilize the CO_2_ molecule through electrostatic interactions and enhance its interaction capabilities. In contrast, cations in solution can compete with the CO_2_ molecule for chemisorption on the catalyst, thereby reducing the efficiency of CO_2_ capture. Zhichao Zhang et al. [34] report that for CO_2_RR, different alkali metal cations interact with CO_2_ under discrepant coordination features, i.e., Li^+^/Na^+^ coordinates with one O atom, whereas K^+^ may coordinate with both O atoms of CO_2_. The large atomic radius of potassium allows its interactions and mobility in solution to influence the additional kinetics of electrochemical CO_2_ activation and reduction reactions. Figure 2 schematically illustrates in‐plane bidentate and out‐of‐plane bidentate CO_2_ adsorption in a liquid medium. Figure S6 shows that after 10 ps of AIMD simulation, the initially optimized out‐of‐plane bidentate CO_2_ converts to an in‐plane bidentate, validating the feasibility of in‐plane bidentate CO_2_ activation.

More importantly, the hydrogenation reaction of *CO_2_ on the Sn─Cu/C_3_N_4_‐K_x_^+^(H_2_O) system in the context of its electrocatalytic reduction forms a conventional carboxylic *COOH intermediate and an unconventional formyl *OCHO intermediate (see Figure 2). The *COOH intermediate actively forms through C─Cu and O─Sn bonds, following mixed coordination, due to the Lewis acidity of the Cu atom and the electronegativity of the O atom, allowing efficient charge transfer and stabilizing the reaction. Contrarily, the *OCHO intermediate enables the bonding of both O atoms with Sn─Cu following bidentate coordination; one oxygen atom interacts with Cu to form a strong bond due to electron back‐donation from Cu into the π* orbital of *OCHO, and the second oxygen atom interacts with Sn, providing additional stabilization through Lewis acid interaction. The direct link of *COOH with Cu may achieve stable bonding, but the direct interaction could limit flexibility in specific reaction pathways; conversely, the utilization of both metals by *OCHO intermediates engages more dynamic interaction, escalating the chance of electron transfer processes, enhancing the reactivity across a broad range of reactions. In the following sections, we focus on the interaction of CO_2_ and corresponding *COOH and *OCHO species on the Sn─Cu/C_3_N_4_‐K_x_^+^(H_2_O) model.

### 2.2 Simulating CO_2_ Adsorption and Initial Protonation

**FIGURE 3:**
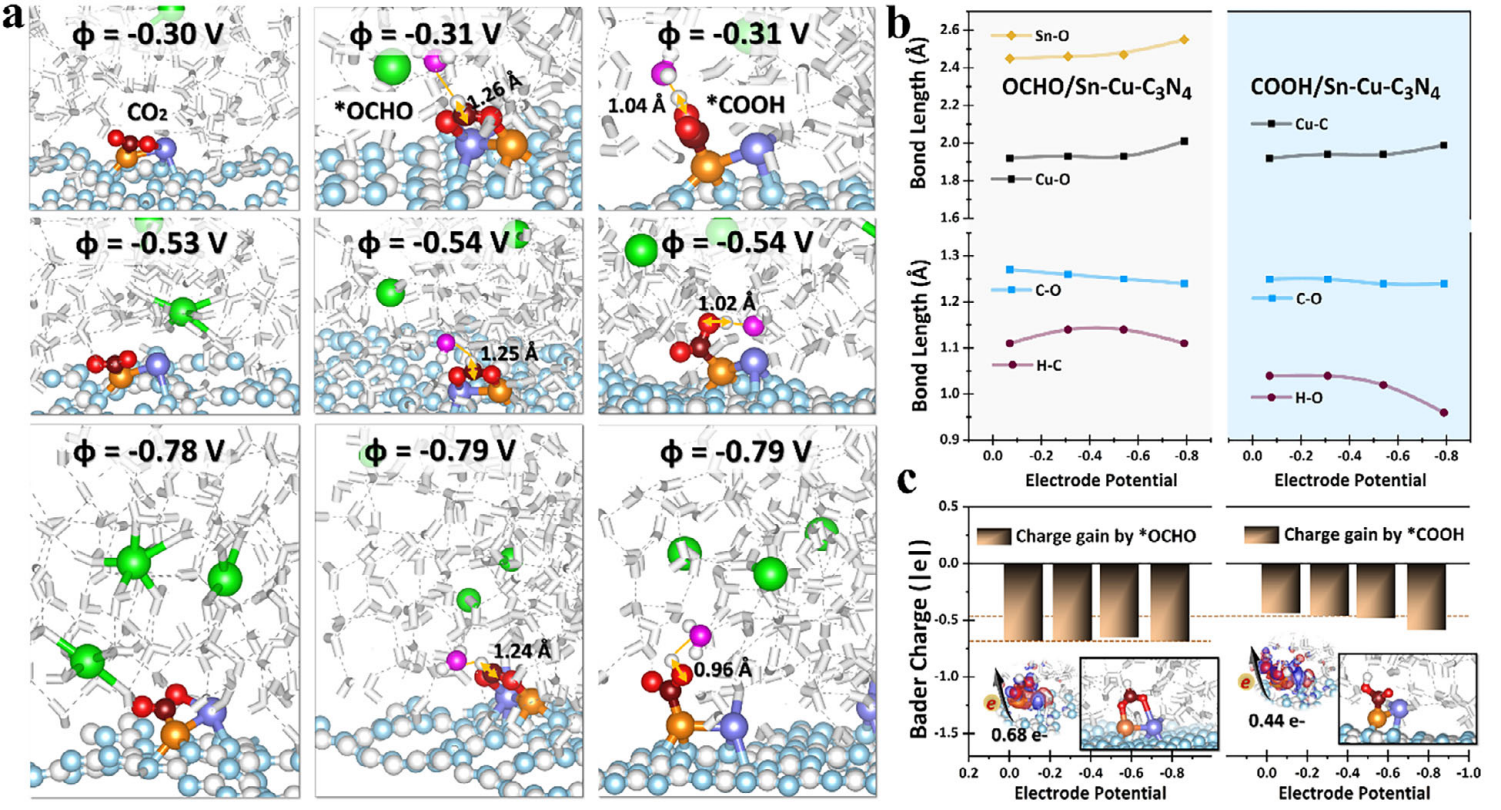
CO_2_ adsorption and protonation at varying electrode potentials: (a) Geometric illustration of *CO_2_ adsorption and protonation into *COOH and *OCHO under three different electrode potentials of 1, 2, and 3 K‐ions. (b) The corresponding bond length deviation of Sn─Cu atoms with O, C atoms of *COOH/*OCHO, and C─O, H─C, and H─O bonds in reaction intermediates. (c) Charge gain by *COOH and *OCHO through Bader charge integration under electrode potentials of −0.31, −0.54, and −0.79 V, respectively. (d) Difference in charge density (Δρ) of *OCHO/*COOH on Sn─Cu/C_3_N_4_ catalyst under a cation‐free environment, Δρ = ρ(OCHO/Sn‐Cu/C_3_N_4_) – ρ(OCHO) – ρ(Sn‐Cu/C_3_N_4_) and Δρ = ρ(COOH/Sn‐Cu/C_3_N_4_) – ρ(COOH) – ρ(Sn‐Cu/C_3_N_4_). Charge depletion and accumulation areas are presented in red and blue colors, respectively, and the value of the iso‐surface is set to be 0.01 e/Å^3^.

It is manifest from prior investigations that the adsorption of the CO_2_ molecule in aqueous solution efficiently influences the selectivity and kinetics of CO_2_RR, making it a focal point for carbon dioxide utilization [35]. In this segment, we thoroughly elucidated the function of the liquid medium, incorporating AIMD simulations for various intermediate stages. In conjunction with the liquid environment, we introduce the factor of electrode potential for the adsorption and protonation of CO_2_ (see Figure 3a). The electrified surface is concocted by acquainting the potassium atoms in a liquid medium with an electrode potential of ∼−0.30 V, ∼−0.53, and ∼−0.78 V, corresponding to increasing numbers of K atoms, respectively. Figure 3a presents the AIMD‐originated trajectories in the context of escalating electrode potentials for *CO_2_ adsorption and its expounded protonation into *OCHO and *COOH intermediary steps. The Sn─O/Cu─O and Cu─C bond lengths in *OCHO and *COOH minutely increase as the strength of the electrode potential increases; however, the C─O bonds show a slight descending pattern in either case. Elevating potentials in an aqueous medium leads to changes in the electron densities and oxidation states of the Sn and Cu metal centers, resulting in minor increases in bond lengths and weakening the Sn─O/Cu─O and Cu─C bonds. Furthermore, *OCHO acquired more negative charges than *COOH, as shown in Figure 3c for liquid and Figure S7 for the vacuum phase, even in a cation‐free model, determining its strong interaction with the Sn─Cu/C_3_N_4_ catalyst.

In addition, the projected crystal orbital Hamilton population (‐pCOHP) analysis in Figure S8 indicates that *OCHO intermediate is more stable chemically, with significant bonding contributions and favorable electronic interactions, whereas *COOH displays relatively weaker bonding interactions and lower stability. Löwdin charge analysis further confirms enhanced charge transfer for *OCHO compared to *COOH, which experiences restricted charge transfer, affecting its reactivity. These findings highlight the critical role of electronic structure in determining the preferred pathway in CO_2_RR and suggest that *OCHO is the dominant intermediate for improved catalytic performance. These results provide valuable insights into the adsorption behavior of these molecules, highlighting the superior stability and charge‐transfer characteristics of *OCHO. Consequently, to attain the potential facilitated by cations for CO_2_ activation and further protonation steps, we spotlighted the free energy profiles for CO_2_ activation and initial hydrogenation on the Sn─Cu/C_3_N_4_ catalyst.

**FIGURE 4:**
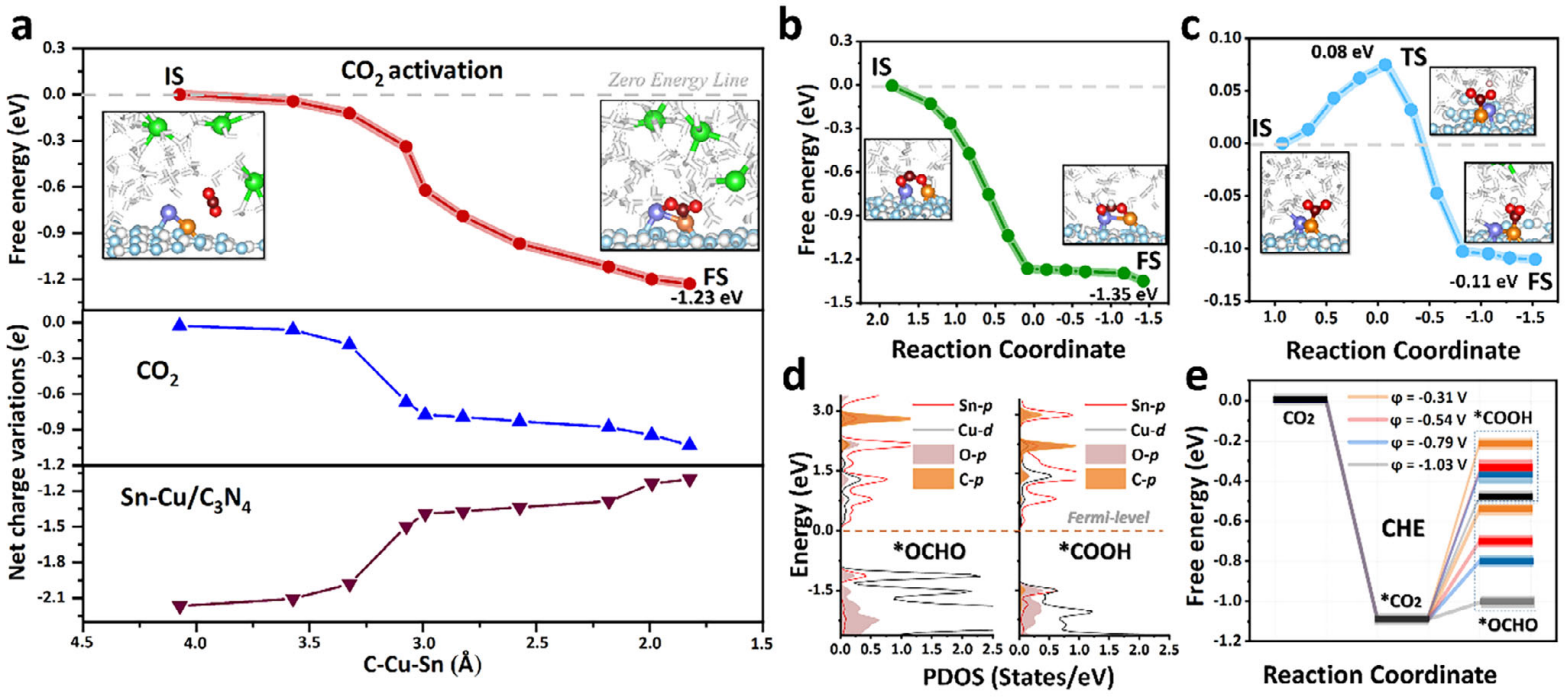
cDFT‐MD simulated free energy profiles for CO_2_ activation and its initial hydrogenation on Sn─Cu/C_3_N_4_ catalyst: (a) Constrained molecular dynamics (cMD) calculated free energy profile and correlated initial state (IS) and final state (FS) geometries for the CO_2_ activation process under −0.78 V electrode potential‐based system, (a gray‐dashed line indicates the zero energy level) the corresponding Bader charge analysis of CO_2_ activation and Sn─Cu/C_3_N_4_ catalyst for each contained point during the constrained molecular dynamics. Free energy profiles and the corresponding structures at IS, FS for (b) *OCHO formation and IS, TS, and FS for (c) *COOH formation on Sn─Cu/C_3_N_4,_ either at an electrode potential of −0.78 V in a liquid medium. (d) The Perdew–Burke–Ernzerhof (PBE) calculated partial density of states (PDOS) after ingesting the *OCHO and *COOH on Sn─Cu/C_3_N_4_ in the liquid medium, with the Fermi level fixed at zero. The *p*‐O state of *OCHO and *p*‐C states of *COOH exist near the Fermi level along with *p*‐Sn and *d*‐Cu orbitals. (e) Juxtapositiong the free energy profiles for the hydrogenation of CO_2_ ensuing *OCHO/*COOH with ascending electrode potential of −0.31, −0.54, −0.79, and −1.03 V in the CHE model.

Figure 4a shows the inner‐sphere charge transfer of CO_2_ with a corresponding free energy profile on the Sn─Cu/C_3_N_4_‐H_2_O system in the presence of a heightened electrode potential, with an exceptionally robust free energy of −1.23 eV. To facilitate the comparative evaluation, we show the free energies of CO_2_ in Figure S9 under the conditions characterized by a low concentration of ions in the system, where the free energy follows a decreasing trend with an ascending concentration of ions; this direction remains steady with a CO_2_RR under cations in the previous report [11]. Beyond that, the comprehended response of CO_2_ adsorption on Sn─Cu/C_3_N_4_ catalyst under a high electric potential is examined by employing the Bader charge from the cMD simulations for every constrained point, as shown in Figure 4b. As the CO_2_ molecule proceeds towards the surface of Sn─Cu/C_3_N_4_, the net charge gained by it gradually approaches a maximum negative value of ∼−0.9 |e|, corroborating that CO_2_ procures amplified electrons. The elevated charge acquired by the carbon dioxide from the Sn─Cu/C_3_N_4_ manifests its chemisorption, which necessitates a more negative electrode potential.

Next, we elucidate the critical phenomena involving the protonation of the CO_2_ molecule and its conversion into *OCHO and *COOH under an acidic condition of CO_2_^−^ + H^+^ → *OCHO/*COOH. Figure 4c,d shows the corresponding cMD simulation results with free energies of −1.23 eV for *OCHO and 0.08 eV for *COOH, indicating that the Sn─Cu/C_3_N_4_‐K_x_+(H_2_O) system possesses a high affinity for converting CO_2_ into a bidentate coordinated *OCHO intermediate. The reactivity of the Sn─Cu/C_3_N_4_ catalyst towards the *OCHO and *COOH intermediate stages is further studied by analyzing their electronic properties. The partial densities of states (PDOS) in Figure 4e show that besides the contribution of Sn and Cu atoms, the contribution of the *p*‐state of oxygens in *OCHO prominently exists near the Fermi level. Driven by the unique electronic configuration of Sn as its *p*‐block metal, it shows a robust bonding interaction with oxygen, identifying that Sn and Cu are vital agents for an energetically favorable interaction with *OCHO. In contrast, the PDOS of *COOH shows a relatively weaker contribution of *p*‐C states near the Fermi level. Our constrained molecular dynamics‐based results identify that the Sn─Cu/C_3_N_4_ catalyst exhibits a higher affinity for *OCHO, directing it to thermodynamically favorable methanol production.

Together with the preceding solvent model, the free energy variation from the CHE model is also analyzed, as shown in Figure 4f. Although the free energy of hydrogenation to *COOH diminishes progressively with increasing electrode potential, the free energy required for the conversion of CO_2_ to *OCHO remains more energetically favorable than that of *COOH formation, even at the lowest potential. These findings indicate that the protonation of the CO_2_ preferentially yields *OCHO over *COOH not only in the liquid phase but also within the confines of the CHE model, thereby elucidating the thermodynamic and kinetic advantages associated with the *OCHO intermediate. Leveraging the effective coordinates of *OCHO through its two oxygen atoms with the dual atoms, the subsequent section explores the further hydrogenation of *OCHO, leading to intermediate stages with significant oxygen interaction with Sn, ultimately forming methanol as the final product.

### 2.3 The Origin of Methanol Production

**FIGURE 5:**
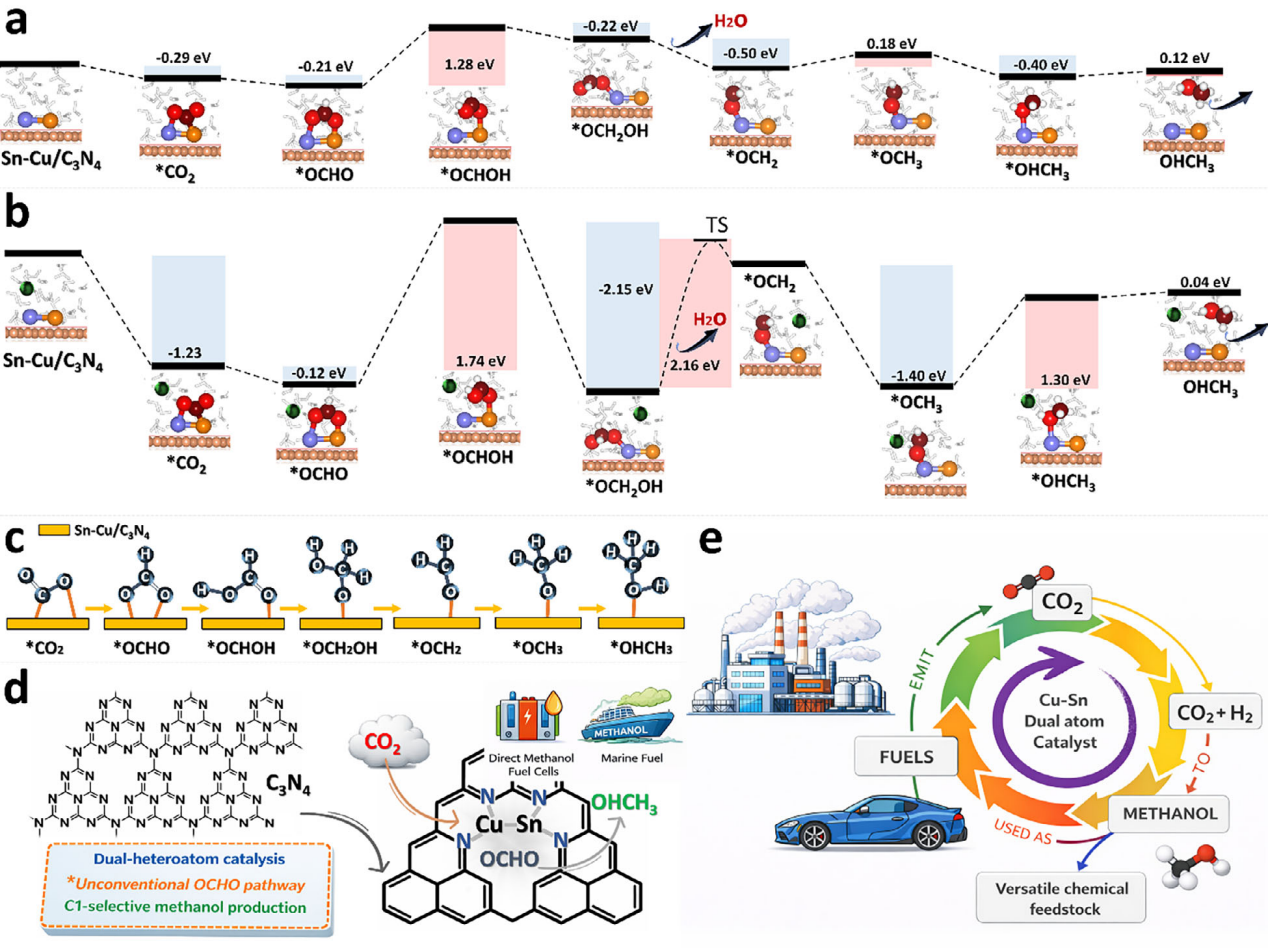
CO_2_ electroconversion into methanol on Sn─Cu/C_3_N_4_ catalyst: (a) Free energy profiles for the CO_2_‐to‐OHCH_3_ on dual‐atom Sn─Cu/C_3_N_4_ catalyst under the electrode potential of (a) −0.09 V using simulated annealing method and (b) −0.79 V cMD simulations in the aqueous solution. CO_2_RR at Sn─Cu/C_3_N_4_ with six stable protonation steps, electroconversion into OHCH_3_ with an atypical *OCHO intermediate. (c) Proposed mechanism of CO_2_ reduction into OHCH_3_ via *OCHO → *OCHOH → *OCH_2_OH → *OCH_2_ → *OCH_3_ → *OHCH_3_ intermediates stages (The asterisk (*) indicates the energetically stable adsorbed states). (d) Chemical structure of Sn─Cu/C_3_N_4_ catalyst. (e) Transforming CO_2_ into useful goods for long‐lasting energy uses.

To further explore the thermodynamically possible reaction pathways on Sn─Cu/C_3_N_4_‐H_2_O, the selectivity for methanol undergoes a pathway with six hydrogenation steps of carbon dioxide. Initially, the free energy for activation of CO_2_ under a low potential is found to be −0.29 eV (see Figure 5a), indicating that CO_2_ adsorbs exothermally. The first protonation of *CO_2_ into *OCHO shows a low energy barrier of −0.21 eV. Further, *OCHO → *OCHOH is the limiting step because the highest energy barrier among all the steps exhibits a limiting potential of 1.28 eV. Earlier studies found activation barriers of 1.02 eV on Ho‐doped Cu(211) [36], 1.06 to 1.19 eV on Ir/In_2_O_3_ [37], and 1.41 to 1.51 eV on Pd surfaces [38], for important methanol‐forming hydrogenation steps. These findings show that barriers above 1 eV often occur in methanol synthesis from CO_2_ and correspond to the kinetically limiting step of the reaction pathway. The identical trend of free energy barrier has also been noticed during the conversion of CO_2_‐to‐OHCH_3_ using the computational hydrogen electrode model (CHE), as illustrated in Figure S10. The preceding reaction steps involving OCH_2_OH, *OCH_2_, *OCH_3_, *OHCH_3,_ and OHCH_3_↑ intermediates show the minute energy barriers of −0.22, −0.50, 0.18, −0.40, and 0.12 eV, respectively. Moreover, pCOHP (see Figure S11) analysis reveals that as the reaction progresses, the bonding interactions between the adsorbate and the catalyst weaken, particularly after the formation of *OCH_3_. This stage marks a transition where desorption becomes energetically favorable. The stabilization of intermediates like *OCHO and *OCHOH is facilitated by the efficient charge transfer from the Sn─Cu/C_3_N_4_ surface, while the transition to *OCH_3_ and its eventual desorption is driven by reduced binding interactions.

The production of methanol is also observed at a higher electrode potential, homogeneous to a low potential. *OCHO remains the key intermediate with a low energy barrier of −0.12 eV, as shown in Figure 5b. For a deeper understanding, the corresponding cMD‐calculated free energy plots include each constraint point for all reaction steps, which include *OCHO → *OCHOH → *OCH_2_OH → *OCH_2_ + H_2_O → *OCH_3_ → *OHCH_3_ → *OHCH_3_↑ intermediate stages are presented in Figure S12. However, the protonation of *OCHO into *OCHOH requires a relatively high energy barrier of 1.74 eV. It is worth emphasizing that the strong oxygen binding with dual Sn─Cu atoms leads to a high reaction barrier in *OCHO → *OCHOH. Further, the *OCHOH → *OCH_2_OH steps exhibit a negative energy barrier of −2.15 eV, clarifying that this step is energetically favorable and exothermic. Next, the protonation of *OCH_2_OH to *OCH_2_ involves an energy barrier of 2.16 eV through the transition state. It is nominated as a limiting step for the liquid system with a maximum electrode potential. Following this, *OCH_2_ is transformed into *OCH_3_ intermediate and is characterized by a reaction barrier of −1.40 eV, showing that this reaction includes an energy release. Finally, the protonation of *OCH_3_ leads to the formation of *OHCH_3_ in the last stage of the reaction, with an energy barrier of 1.30 eV, essentially completing the reaction and culminating in the production of methanol, which can be further detached from the catalyst with a minor energy barrier of 0.04 eV. Though the CO_2_ activation and initial protonation into *OCHO are more favorable on the dual‐atom Sn─Cu/C_3_N_4_ catalyst under a high electrode potential in the aqueous solution, the low limiting potential and the energetically stable hydrogenation steps at low potential lead to the efficient production of methanol.

The reduction of the CO_2_ molecule on dual‐atom Sn─Cu/C_3_N_4_ involves the formate as a key intermediate stage through active bidentate coordination of *OCHO with Cu and Sn atoms; further protonation energetically favors the single‐carbon (C_1_) methanol. Typically, the protonation of *COOH intermediate leads to *CO, which is considered to be thermodynamically more feasible for two‐carbon products [39]. However, barrier energies are more stabilized for the *OCHO intermediate, which dominates the methanol as a product. On Sn─Cu/C_3_N_4_, the formation of the C_1_ product is energetically favored compared to the long‐chained C_2_ product, i.e., the formation of ethanol requires concern about how the C─O cleavage is protected from protonation [40]. The combination of Sn─Cu selectively promotes the single‐carbon species, including forming C─H and C─O bonds, while destabilizing the transition steps required for C─C coupling, forming multicarbon compounds. Based on our calculations and discussion, we conclude that the preliminary protonation of CO_2_ forms monodentate‐coordinated *COOH and bidentate‐coordinated *OCHO species, where the *OCHO is presumably the pivotal intermediate in producing methanol.

## 3 Discussion

We have studied the unique bidentate coordination and thermodynamics of the *OCHO intermediate and their importance for the kinetics of CO_2_‐to‐OHCH_3_, assumed to be the most substantial reaction step for CO_2_RR on hetero‐diatomic Sn─Cu/C_3_N_4_ catalyst, as schematically summarized in Figure 5c,d. From the outset, the activation of a CO_2_ molecule in a water environment in the presence of potassium cations has a low free energy of −1.23 eV. At the same time, the low concentrations of K‐ions show relatively higher free energies for CO_2_ activation (−0.66 and −1.10 for 1K^+^ and 2K^+^, respectively). During CO_2_ activation via constrained cMD simulations, as the CO_2_ molecule approaches the Sn─Cu/C_3_N_4_ surface at each constrained point, the charge gain by CO_2_ gradually increases and reaches a more negative value. This confirms the strengthened chemisorption of CO_2_ on the Sn─Cu/C_3_N_4_ catalyst because of the negative electrode potential. Besides investigating the effect of cations on CO_2_ activation in an aqueous medium using a diatomic Sn─Cu/C_3_N_4_ catalyst, we also analyzed the protonation of CO_2_ in this environment. Our cMD results led to two intermediate stages, namely *COOH and *OCHO, in an acidic CO_2_^−^ + H^+^ → *OCHO/*COOH condition with free energies of −1.35 and 0.08 eV. These results justify that dual‐atom Sn─Cu efficiently converts *CO_2_ into *OCHO. The PDOS visualizes the significance of p‐O states in *OCHO, leading to more charge gain. The *p*‐states of the Sn atom in the dual atom effectively interact with an oxygen atom.

Both atoms in *OCHO make bonds with Sn and Cu atoms of the catalyst; the bonding of Cu with O originates from donating an electron from Cu to the π* orbital of *OCHO, and the Sn interacts with O atoms through Lewis acid interaction. On the other hand, the direct bonding of the C atom of *COOH with Cu may achieve high stability while possibly reducing the flexibility of various reactions. Our DFT and cMD simulation results show that the protonation of CO_2_ towards the unconventional oxygen‐coordinated *OCHO intermediate is more energetically favorable than the *COOH intermediate. To further support our argument regarding the importance of the *OCHO intermediate, we also investigated the free energy difference using the CHE model. The free energy barrier for the protonation of CO_2_ into *COOH gradually reduces with the ascending electrode potentials, but a considerable energy barrier still exists even at a very high electrode potential. On the contrary, the energy barriers for *OCHO are comparatively low even at the lowest possible potential in the CHE model. We, therefore, conclude that *OCHO is chemically preferable in both CHE and aqueous medium on the Sn─Cu/C_3_N_4_ surface. Next, the protonation of *OCHO is explored to unveil the possible thermodynamic reaction steps for methanol formation using simulated annealing and cMD simulations. The generation of methanol involves six stepwise protonations by considering the following equations: CO_2_^−^ + 6H^+^ → OHCH_3_ + H_2_O. After forming *OCHO, the second intermediate *OCHOH is formed through an endothermic reaction with a high energy barrier of 1.28 eV. Further hydrogenation of *OCHOH leads to *CHO, *OCH_2_, *OCH_3_, and OHCH_3_ with moderate energy barriers of −0.22, 0.50, 0.18, and −0.40 eV, respectively. The reduction reaction of CO_2_ on Sn─Cu/C_3_N_4_ through the *OCHO, consisting of bidentate coordination with Cu and Sn, is the key intermediate for methanol products. In addition, the thermodynamically stable formation of the C1 product on Sn─Cu/C_3_N_4_ is verified by the fact that, for the C_2_ product, for example, ethanol is only formed when C─O cleavage is not involved in the protonation. The low probability of *COOH typically leads to *CO restraining the formation of two‐carbon products. While the CO pathway has the lowest limiting potential (−0.25 V) (see Figure S13), the strong stabilization of the *OCHO intermediate (Δ*G* ≈ −0.65 eV) and its hydrogenated derivatives supports a viable formate‐based route to CH_3_OH formation in the gas phase. These findings show that CO_2_RR selectivity depends not only on limiting potentials but also on the stability of reaction intermediates and the balance between desorption and further hydrogenation. The diatomic Sn─Cu supports form C_1_ products consisting of reinforced C─H and C─O bonds and avoids the steps necessary for C─C coupling.

## 4 Conclusion

In conclusion, we have used DFT and cMD simulations to reveal the importance of the uncommon *OCHO intermediate needed for CO_2_ reaction steps on the dual‐atom Sn‐Cu/C_3_N_4_ catalyst─water interface under the electrode potential of ions. Primarily, we identified that electrode potential facilitates the activation of CO_2_ at the Sn─Cu/C_3_N_4_‐water interface, achieving a low free energy of −1.23 eV with a substantial charge gain of approximately −0.9 |e|. Our findings demonstrate that the initial protonation of CO_2_ results in the formation of two intermediates: *COOH, wherein carbon is bonded to Cu, and *OCHO, characterized by two oxygen atoms bonded to diatomic Sn─Cu. The bidentate coordination *OCHO intermediates with Sn─Cu atoms instigates further reduction reactions on the Sn─Cu/C_3_N_4_ surface; the six protonation steps of CO_2_ through the most critical *OCHO intermediate form methanol as a product. Overall, cMD simulations unveil the molecular‐level understanding of dual heteroatom catalysts for CO_2_RR and modulate the non‐traditional *OCHO intermediate, which potentially stimulates the origin of methanol formation.

## 5 Method

### 5.1 Computational Details

All simulations use density functional theory through the Vienna Ab initio Simulations Package (VASP) [41], while employing the projector augmented‐wave [42, 43, 44]. We have used Perdew–Burke–Ernzerhof within the generalized gradient approximation for the electron exchange–correlation interactions [45]. The PBE exchange–correlation functional is employed because it has been widely used and validated in recent DFT studies of CO_2_ adsorption on catalytic surfaces, providing a reasonable balance between computational efficiency and accuracy for predicting adsorption geometries and energetics [28]. The convergence criteria for total energy as 10^−4^ eV/Å and Hellmann‐Feynman forces as 0.01 eV/Å are considered for structural relaxation. The cutoff energy is set to 400 eV, and the Brillouin zone is used for setting the *k*‐mesh of 6 × 6 × 6, and the Bader charge integration is adopted to determine the charge exchange during the CO_2_ activation and reduction reactions [46, 47, 48]. Grimme (DFT‐D3 method) [49], is used to correct van der Waals interactions within our system. LOBSTER code is employed to compute ‐pCOHP and Löwdin charge analysis, providing insights into bonding character and charge transfer.

The hydrogen electrode (CHE) model proposed by Nørskov et al. [50] is used, which can be expressed by Equation (1):

(1) $$\Delta G=\Delta E+\Delta ZPE-T\Delta S+eU+k_{B}T\;\ln10*\left(\mathrm{pH}\right)$$

where *ΔE* is the chemical reaction energy; *ΔZPE* and *TΔS* correspond to the correction of zero‐point energy and harmonic entropy, respectively, and *U* is the term used for applied potential.

### 5.2 Computational Model of Dual Atom Sn─Cu/C_3_N_4_ Catalyst–Water Interface

Molecular‐level simulations are performed to analyze the interactions between CO_2_ activation and hydrogenation at the Sn─Cu/C_3_N_4_ catalyst–water interface, and the effect of the electrode potential is accounted for by introducing potassium atoms into the system. The model consists of a 2 × 2 × 1 supercell of C_3_N_4_ and 88 H_2_O molecules, interface along the *z*‐direction at 1 atm pressure. Cations are positioned at the Sn─Cu/C_3_N_4_‐water interfacial models, thereby designing the Sn─Cu/C_3_N_4_‐water‐K*_x_* (*x* = 1–3). AIMD simulations evaluate the kinetics of CO_2_ activation on the Sn─Cu/C_3_N_4_‐water‐K*_x_* interface. During AIMD simulations, the Nose‐Hoover thermostat maintains the canonical ensemble (NVT) at 300 K. Standard 10 ps AIMD simulations are performed for the Sn─Cu/C_3_N_4_‐water‐K*_x_* interface, allowing the water molecules to approach equilibrium. Electrode potential is tuned by altering the potassium ion concentration, which, in turn, varies the interfacial charge, thereby regulating the electrode potential [51]. The electrode potential (Φ) is found using Equation (2).

(2) $$\Phi =\frac{\sigma }{C}+\Phi_{\mathrm{PZC}}$$

In Equation (2), σ is the total surface charge, C is the interfacial capacitance (∼21 µF/cm^2^) [52, 53], and ΦPZC is the potential of zero charge (−0.07 V vs. RHE) of the graphene sheet.

### 5.3 Constrained Molecular Dynamics (cMD)

To calculate free energy profiles and kinetic barriers for CO_2_ activation and hydrogenation, we use cMD and the thermal integration method [54]. This technique introduces a holonomic constraint on the coordinate of reactions along activation and hydrogenation paths. During the thermodynamics integration over solvent, the average forces of constraint for the particular reaction coordinate (ζ), we have computed the free energy concerning the chosen reference state, which can be represented according to the given Equation (3):

(3) $$\Delta A\;\left(\zeta \right)=-\int_{\zeta_{a}}^{\zeta_{b}}F\left(\zeta \right)d\zeta$$

In Equation (3), ΔA(ζ) shows the free energy difference between two reaction coordinates, i.e., ζa and ζb, and F(ζ) is the average force of the constrained reaction coordinate. During the activation of CO_2_ on the dual‐atom Sn─Cu, the average distance between Sn─Cu and the C atom of CO_2_ is defined as a collective variable (CV). The CV for the other hydrogenation steps, for instance, the formation of *OCHO, is determined by considering the H─O distance in the H_2_O molecule and the distance of the H atom of H_2_O from the C atom of CO_2_ (see Figure S14).

## Conflicts of Interest

The authors declare no conflicts of interest.

## Supporting information




**Supporting File**: smtd70874‐sup‐0001‐SuppMat.docx.

## Data Availability

The data that support the findings of this study are available from the corresponding author upon reasonable request.
