# Supplementary material for: Selective Production of Methanol via Bidentate Coordination of an Intermediate on the Dual‐Atom Electro‐Catalyst
Source: Small Methods. 2026 Jul 28;10(16):e70874. doi: 10.1002/smtd.70874 (PMC13505753; doi:10.1002/smtd.70874)
Supplement: Supplementary file 1 [file SMTD-10-e70874-s001.docx]

Supporting Information

Selective Production of Methanol via Bidentate Coordination of Intermediate on the Dual-atom Electro-Catalyst

*Imran Muhammad,^1^*, Danish Khan,^2^ Tanveer Hussain,^3^ Khuram Walayat,^4^ Awais Ghani,^5^ Kemeng Ji,^6^ Shehzad Ahmed,^7^* Asif Mahmood,^8^**

**Contents**

[1. Atomic structure of dual-atom Sn-Cu/C_3_N_4_ catalyst	2](#_Toc68184715)

2. Electronic properties of CO_2_@Sn-Cu/C_3_N_4_	3

[3. Model of dual-atom Sn-Cu/C_3_N_4_ catalyst in liquid medium	3](#_Toc68184716)

[4. In-plan and out-of-plan bidentate CO_2_ adsoprtion	4](#_Toc68184716)

[5. Charge distribution for CO_2_ and initail intermediates	5](#_Toc68184717)

[6. –pCOHP and Löwdin charge analysis for *COOH/*OCHO	6](#_Toc68184717)

[7. cMD plots for CO_2_ activation at different electrode potentials	7](#_Toc68184718)

[8. CHE calculated hydrogenation stages for methanol production	8](#_Toc68184718)

[9. –COHP for *CO_2_ and six protonantion stages	9](#_Toc68184719)

[10. cMD simulated plots for free energy profiles during CO_2_RR	10](#_Toc68184720)

[11. CO_2_RR paths on Sn-Cu/C_3_N_4_	12](#_Toc68184720)

[12. Selection of collective variables using reaction coordinates	13](#_Toc68184721)

1. **Atomic structure of dual-atom Sn-Cu/C_3_N_4_ catalyst**

**Figure. S1** Top and side view of a relaxed supercell of C_3_N_4_ nanosheet, dual atom Sn and Cu are adsorbed on the C_3_N_4_ sheet bonded with N atom, with vacuum along the *z*-direction of the cell are set to be 20 Å. In the dual atom Sn-Cu C_3_N_4_ catalyst, Sn and Cu atoms are bonded with a minute bond length of 2.48 Å, and the Cu atom is bonded with N atoms of catalysts with a bond distance of 2.09 and 1.98 Å. In contrast, Sn atom makes a relatively longer bond length of 2.34 and 2.36 Å with the N atoms of the catalyst. (Here, light blue, white, golden, and purple spheres represent the nitrogen, carbon, copper, and tin atoms, respectively.

2. **Electronic properties of CO_2_ adsorbed on dual-atom Sn-Cu/C_3_N_4_ catalyst in gas phase**

**Figure S2.** Projected density of states (PDOS) for the Sn–Cu/C_3_N_4_ catalyst before and after CO_2_ adsorption in the gas phase.

**Figure S3.** Crystal orbital Hamilton population (COHP) analysis of key C/O–metal interactions after CO_2_ adsorption. Negative ICOHP values (C–Cu: –2.39 eV, C–Sn: –1.33 eV, Cu–O: –1.18 eV, Sn–O: –1.10 eV) indicate bonding contributions, with the C–Cu bond (2.06 Å) showing the strongest stabilization.

**Figure S4.** Projected density of states (PDOS) for Cu 3d orbitals before and after CO_2_ adsorption. The occupied Cu d-band center shifts from −2.76 eV to −3.05 eV upon CO_2_ binding, indicating redistribution of d-electrons and confirming electronic interaction between the Sn–Cu dual-metal site and CO_2_.

3. **Model of dual-atom Sn-Cu/C_3_N_4_ catalyst in liquid medium**

**Figure S5.** **a** The side and **b** top views of a 14.20 Å × 14.20 Å × 20.0 Å supercell of dual atom Sn-Cu/C_3_N_4_ model with a liquid environment of pure water. The fluctuation plot of water density distribution along the *z-*direction in the Sn-Cu/C_3_N_4_-pure-water model is shown in **a**; this analysis is extracted from the final two picoseconds AIMD simulations trajectory.

4. **In-plan and out-of-plan bidentate CO_2_ adsoprtion**

**Figure S6.** The optimization of **a** in-plane bidentate and **b** out-of-plane bidentate CO_2_ adsorption on dual-atom Sn-Cu/C_3_N_4_ under an aqueous medium. (The out-of-plane bidentate adsorption in **b** converts into in-plane bidentate adsorption after a 10 psec AIMD simulation)

5. **Charge distribution for CO_2_ and initial hydrogenated states**

**Figure S7.** Visualization of charge density difference of adsorbed **a** CO_2_ molecule and its initial hydrogenation states **b** *COOH and **c** *OCHO on dual-atom Sn-Cu/C_3_N_4_ catalyst in a vacuum, where green and brown colors show the holes and electrons at an isosurface value of 0.02 e/Å^3^.

6. **-pCOHP and Löwdin charge** **analysis and of *COOH and *OCHO intermediates**

**Figure S8.** The calculated -pCOHP results for *COOH and *OCHO, along with Löwdin charge analysis, indicate that *OCHO is more stable with enhanced charge transfer compared to *COOH, which exhibits limited charge transfer and reduced structural stability as reflected in the -pCOHP analysis.

7. **cMD plots for CO_2_ activation at different electrode potential**

**Figure S9.** Constrained molecular dynamics (cMD) calculated the free energy profile and correlated initial state (IS) and final state (FS) geometries for the CO_2_ activation process under **a** -0.09 V, **b** -0.31, and **c** -0.54 V electrode potential.

8. **CHE calculated hydrogenation stages for methanol production**

**Figure S10.** Free energy profile of CO_2_-to-OHCH_3_ on dual-atom Sn-Cu/C_3_N_4_ using CHE model in a clean system.

9. **-COHP for *CO_2_ and 6 protonation stages**

**Figure S11.** The -COHP for *CO_2_ and 6 protonation stages *OCHO → *OCHOH → *OCH_2_OH → *OCH_2_ → *OCH_3_ → *OHCH_2_ →*OHCH_3_.

10. **cMD simulated plots for free energy profiles during CO_2_RR**

**Figure S12.** Constrained molecular dynamics (cMD) simulated free energy profiles for the hydrogenation steps of CO_2_ and the detachment of OHCH_3_ from the catalyst under -0.79 V electrode potential on the Sn-Cu/C_3_N_4_. The hydrogenations includes *OCHO → *OCHOH → *OCH_2_OH → *OCH_2_ + H_2_O → *OCH_3_ → *OHCH_2_ → *OHCH_3_↑ intermediates stages. The initial and final stages of all the reactions are shown geometrically.

11. **CO_2_ adsorption and hydrogenation, following paths on the Sn-Cu/C_3_N_4_ catalyst**

**Figure S13.** Geometric structures of CO_2_ adsorption and intermediate species after hydrogenation, following paths 1, 2, and 3 on the Sn-Cu/C_3_N_4_ catalyst.

**12. Selection of collective variables using reaction coordinates**

**Figure S14.** The geometric representation of CV is used as reaction coordinates while employing the constrained MD method on the Sn-Cu/C_3_N_4_ in aqueous conditions for CO_2_ activation, and formation of *OCHO and **c** *COOH species.
